# Progress in High-Amylose Cereal Crops through Inactivation of Starch Branching Enzymes

**DOI:** 10.3389/fpls.2017.00469

**Published:** 2017-04-04

**Authors:** Juan Wang, Pan Hu, Zichun Chen, Qiaoquan Liu, Cunxu Wei

**Affiliations:** ^1^Jiangsu Key Laboratory of Crop Genetics and Physiology/Co-Innovation Center for Modern Production Technology of Grain Crops, Yangzhou UniversityYangzhou, China; ^2^Joint International Research Laboratory of Agriculture and Agri-Product Safety, Yangzhou UniversityYangzhou, China

**Keywords:** high-amylose cereal crop, starch branching enzyme, starch molecular structure, starch property, heterogeneous starch granule

## Abstract

High-amylose cereal starches provide many health benefits for humans. The inhibition or mutation of starch branching enzyme (SBE) genes is an effective method to develop high-amylose cereal crops. This review summarizes the development of high-amylose cereal crops through the inactivation of one or more SBE isoforms or combination with other genes. This review also reveals the causes of increase in amylose content in high-amylose crops. A series of changes, including amylopectin structure, crystalline structure, thermal properties, and hydrolysis properties, occurs as amylose content increases. The different morphological starch granules nominated as heterogeneous starch granules or differently stained starch granules are detected in high-amylose cereal crops. Detailed studies on four heterogeneous starch granules in high-amylose rice, which is developed by antisense RNA inhibition of SBEI/IIb, indicate that granules with different morphologies possess various molecular structures and physicochemical and functional properties. This variation diversifies their applications in food and non-food industries. However, current knowledge regarding how these heterogeneous starch granules form and why they exhibit regional distribution in endosperm remain largely unknown.

## Introduction

High-amylose starches, rich in resistant starch (RS), have been extensively investigated because of their many potential health benefits. RS is a portion of starch that can evade degradation in the upper gastrointestinal tract and function as a substrate for the bacterial fermentation in the large intestine (Nugent, 2005). RS-enriched food can decrease glycemic and insulin responses and reduce the risk of developing type II diabetes mellitus, obesity, and cardiovascular diseases (Granfeldt et al., 1995; Regina et al., 2006; Zhu et al., 2012). The proportion of amylose is positively related to RS content (Sang et al., 2008; Cai et al., 2015; Lin et al., 2016b; Zhou et al., 2016). High-amylose starches exhibit various physicochemical properties, including high gelling strength, excellent film-forming ability, and ease of retrogradation; with these properties, industrial applications, such as adhesive, paper, and biodegradable plastic production, have been developed (Avella et al., 2002). Therefore, considerable research has focused on high-amylose starches.

Starch is composed of approximately 15–25% linear amylose. The remaining portion of starch consists of highly branched and organized amylopectin (Smith et al., 2002). Amylose in the endosperm can only be synthesized by granule bound starch synthase I (GBSSI), which is tightly bound to starch granules to extend α-1,4 linkages of glucose polymers (Jeon et al., 2010). Amylopectin synthesis is mainly attributed to three biosynthetic enzymes, namely, starch synthases (SSs), starch branching enzymes (SBEs), and debranching enzymes (DBEs). SSs and SBEs are responsible for the elongation of glucan chains and the formation of branch points, respectively (Jeon et al., 2010; Tetlow and Emes, 2014). The irregular glucan structure produced by SSs and SBEs is trimmed by DBEs to derive an ordered amylopectin branched chains (Nakamura, 2002). Plastidial phosphorylase (Pho1) also participates in starch synthesis; however, its precise role remains unclear (Yu et al., 2001; Satoh et al., 2008).

The amylose content (AC) in cereal endosperm can be effectively increased by applying two methods, that is, enhancing GBSSI expression or eliminating SBEs, SSIIa, or other enzymes involved in amylopectin synthesis (Umemoto and Terashima, 2002; Itoh et al., 2003; Crofts et al., 2012; Zhou et al., 2016). Once a certain level is reached, AC no longer increases in GBSSI-enhanced lines possibly because of the limited non-reducing ends in amylose and the substrate competition between amylose and amylopectin (Tsai et al., 1970; Smith et al., 2002; Sestili et al., 2012). The promotion of AC in SSIIa nulls varies widely in different species; for instance, the elimination of SSIIa unlikely change AC in rice *alk* (Umemoto et al., 2004, 2008), moderately increases AC (40–50%) in maize *sugary2* (*su2*) and wheat *sgp-1* (Yamamori et al., 2000; Tziotis et al., 2004; Zhang et al., 2004), and yields about 50% AC in barley *sex6* (Morell et al., 2003). Compared with the effectiveness of SBE inactivation for high-amylose cereal crops, which have remarkable increase in AC (Li et al., 2008; Carciofi et al., 2012; Zhu et al., 2012; Regina et al., 2015), the enhanced AC in SSIIa nulls is moderate (Yamamori et al., 2000; Tziotis et al., 2004; Zhang et al., 2004). Therefore, AC can be promoted effectively by suppressing or eliminating one or more SBE activities in cereal crops (Table 1).

**Table 1:** **The inactivated gene, amylose content in starch, and starch crystallinity of high-amylose cereal crops**.

**Species**	**The original AC in starch**	**The inactivated genes**		**Name of high AC line**	**AC in starch^b^**	**Starch crystallinity**	**References**
		**Mutation**	**Down regulation^a^**				
Maize	25–30%	SBEIIb		H99*ae*,OH43*ae*, B89*ae*, B84*ae*	61.7–67.7% (I)	B	Li et al., 2008
		SBEIIb (HAM)		GEMS-0067	88.2% (G)	B	Jiang et al., 2010b
Rice	*Japonica* 15.4–19.6%	SBEIIb		EM10	26.5% (I)	B	Nishi et al., 2001
			SBEIIb (~80%)	ami-BEIIb	41.2% (I)	B	Butardo et al., 2011
			SBEIIb (~50%)	hp-BEIIb	34% (I)	A or C	Butardo et al., 2011
			unknown	Goami 2(G2)	33.96% (I)	B	Kang et al., 2003
		SSIIIa		*ss3a*	24.8% (G)	A	Fujita et al., 2007
		SBEIIb, SSIIIa		*ss3a/be2b*	45% (G)	B	Asai et al., 2014
	*Indica* about 25%	SSIIIa		*b10*	~34% (I)	A	Zhou et al., 2016
			SBEI (~100%), SBEIIb (>90%)	TRS	64.8% (I)	C	Zhu et al., 2012
Wheat	22.9–32.3%	SBEIIa (Tilling)		SBEIIa Mutant	55.7% (K)	–	Slade et al., 2012
			SBEIIa (most), SBEIIb (most)	hp-SBEIIa	74.4% (G)	–	Regina et al., 2006
		SBEIIa, SBEIIb		CS2-F11	86.6% (G)	–	Regina et al., 2015
Barley	29.9–31.6%		SBEIIa (>80%)	SBEIIa^−^	38% (G)	–	Regina et al., 2010
			SBEIIa (>90%), SBEIIb (>80%)	SBEIIa^−^/ SBE IIb↓	67.2% (G)	–	Regina et al., 2010
			SBEIIa (>90%), SBEIIb (>95%)	SBEIIa^−^/ SBEIIb^−^	76.2% (G)	–	Regina et al., 2010
			SBEI (87%), SBEIIa (73%), SBEIIb (74%)	SBE RNAi4.1	99.1% (G)	B	Carciofi et al., 2012

a *The content of parenthesis means the reduced expression of targeted genes*.

b *The AC determined by iodine colorimetry, GPC, and K-AMYL kit method is labeled as (I), (G), and (K), respectively*.

This review summarizes the development of high-amylose cereal crops through the inactivation of SBE(s) and a series of starch changes caused by the increased amylose percentage.

## Development of high-amylose cereal crops through SBE inactivation

Cereal crops have three SBE classes, namely, SBEI, SBEIIa, and SBEIIb. Biochemical observations indicate that SBEI preferentially branches longer chains, whereas SBEII isoforms have a higher capacity for transferring of short chains (Guan and Preiss, 1993; Takeda et al., 1993; Guan et al., 1997; Nishi et al., 2001; Nakamura, 2002; Nakamura et al., 2010; Tetlow and Emes, 2014). Among these observations, studies on eliminating SBEI activity show no effect on the starch composition and kernel morphology (Blauth et al., 2002; Satoh et al., 2003b; Regina et al., 2004); however, in rice and maize, minor changes in the fine structure of amylopectin have been reported (Fujita et al., 2006; Xia et al., 2011). However, AC resulting from the inactivation of either one of two SBEII isoforms and the combination of two or three SBE isoforms is significantly increased in different species (Table 1).

## Amylose increase through SBEIIa or SBEIIb deficiency

In maize and rice, SBEIIb inactivation is required to obtain a high-amylose phenotype, whereas no significant changes including AC and amylopectin fine structure are observed in SBEIIa-defective endosperms (Blauth et al., 2001; Nakamura, 2002; Satoh et al., 2003a). Mutations of *SBEIIb* in maize and rice are commonly referred to as *amylose-extender* (*ae*) mutants. Normal maize occupies about 25–30% AC, whereas the AC in H99*ae*, OH43*ae*, B89*ae*, and B84*ae* inbred *ae*-lines has been reported to reach at least 60%, ranging from 61.7 to 67.7% (Li et al., 2008). In rice containing Wx^b^, which mutates at the 5′ end of intron one, *japonica* type contains low GBSSI mRNA and protein levels, leading to reduced AC in contrast to *indica* type, which has a highly expressed Wx^a^ allele (Isshiki et al., 2000). The *ae* mutants from these two backgrounds have higher AC than their wild-type parents. However, AC increase extent is not more than 15%, which is significantly lower than that in analogous maize mutants, which show at least 35% promotion (Nishi et al., 2001; Shannon et al., 2009). Another *ae*-like mutant Goami 2 in rice, also known as Suweon 464, which is derived from high-quality temperate *japonica* rice variety (Iipumbyeo), shows about 2-fold increase in AC (to around 34.0%, see Kang et al., 2003). However, the specific mutation responsible for high-amylose phenotype of Goami 2 remains to be proved.

On the contrary, an almost complete *SBEIIb* inhibition in wheat and barley causes a minor change in AC, whereas *SBEIIa* inhibition can evidently increase AC (Regina et al., 2006, 2010). By targeting induced local lesions in genomes (TILLING), wheat *SBEIIa* mutant increases AC from 23 to 55% (Slade et al., 2012). In barley transgenic lines, by RNA-mediated silencing technology, a high-amylose phenotype (>38%) is observed when SBEIIa expression is reduced by >80% (Regina et al., 2010).

In summary, SBEIIb in rice and maize plays a distinct role in starch synthesis. By contrast, SBEIIa in wheat and barley is more significant than SBEIIb. High-amylose phenotype generation is determined by the difference in SBEIIa and SBEIIb expression levels in cereals. In maize, SBEIIb, which is at least 50 times the abundance of SBEIIa, is the predominant isoform in endosperm (Gao et al., 1997). On the other hand, in rice, the ratio of SBEIIb and SBEIIa is closer to 5:1, which partly elucidates the higher AC promotion in maize *ae* mutants. Furthermore, SBEIIa is expressed at much higher levels than SBEIIb counterpart in both wheat and barley kernels (Regina et al., 2006, 2010). Therefore, SBEIIa inhibition is more effective for developing high-amylose species in the two crops.

## Higher amylose increase through inactivation of two or more SBEs

Combining reduced SBE isoforms with the base of single SBEIIa or SBEIIb inactivation is an efficient way to acquire high-amylose crops, especially for wheat and barley (Table 1). A transgenic resistant starch rice line (TRS) with 60% AC is developed by simultaneously declining the expressions of SBEIIb and SBEI in an *indica* rice variety (Te-qing, TQ). The AC of TRS is significantly higher than that of TQ-derived rice line by declining the expression of SBEIIb alone (Wei et al., 2010c; Zhu et al., 2012). In wheat, transformation with a hairpin construct targeting *SBEIIa* alone not only reduces transcription to <10%, but also leads to an almost complete loss of SBEIIb in the protein levels, which yields an AC of ~75% in transgenic wheat (Regina et al., 2006). Furthermore, a genetic strategy to combine deletion and single nucleotide polymorphism (SNP) generates wheat genotypes with the complete absence of SBEIIa from all three genomes and the absence of SBEIIb from one of the genomes, which elevates the AC to an unprecedented ~85% (Regina et al., 2015). In barley, a combination of SBEIIa and SBEIIb inhibition leads to an amylose increase up to 76.2% at maximum, which is much higher than the suppression by SBEIIa alone (Regina et al., 2010, 2012). In addition, an “amylose-only barley” is derived through concerted repression of SBEI, SBEIIa, and SBEIIb using a chimeric RNAi hairpin (Carciofi et al., 2012). Through this method, the highest known AC-contained lines in rice, wheat, and barley are derived (Table 1).

## Higher amylose increase through SBEII isoform deficiency with other genes

By introducing a modifier gene or by coupling with isozyme deficiencies related to starch biosynthesis on the base of inactivation of SBEII, additive effects show a more profound meaning on AC in cereal crops (Wu et al., 2009; Jiang et al., 2010b; Asai et al., 2014).

When an unknown number of high-amylose modifier (HAM) gene is introduced into a homozygous *ae*-mutant background in maize, AC is further increased (Wu et al., 2009). The commercial maize GEMS-0067, which is the highest known AC-contained line (>85%), is derived from the predigree of [GUAT209:S13 × (OH43ae × H99ae)] (Li et al., 2008; Wu et al., 2009). GUAT209:S13 is a cross-breed serving as HAM gene. Jiang et al. (2010b) found that HAM gene dosage has an additive effect on AC; amylose percentage ranges from 68.9 to 88.2%, when the HAM gene-dosage levels are 0, 16.7, 33.3, 50, 66.7, 83.3, and 100%.

Single SBEII mutation coupled with other high-amylose mutation is an effective method in producing high-amylose starches. SSIIIa deficiency in rice produces 30.7% AC phenotype. However, when this mutation is introduced into SBEIIb-defective background, the AC is further promoted into 45% in *ss3a*/*sbe2b* double mutant (Asai et al., 2014).

## Factors affecting amylose increase in high-amylose cereal crops

Three factors are proposed to be responsible for amylose enrichment caused by SBE isoform lesions in endosperm starch. First, amylopectin synthesis reduction emphasizes the amylose percentage in these high-amylose lines compared with the normal ones. Second, the increase protein amount of GBSSI or higher GBSSI activity results in an elevated amylose synthesis. Third, the extra-long chains (ELCs) resulting from amylopectin, which can bind iodine to develop a dark-blue color, significantly enriches AC value in high-amylose varieties, (Jane et al., 1999). Factors responsible for the amylose increase are different across various species.

In rice, different researchers have various opinions on amylose enrichment. An increase in amylose is attributed to the first two factors. In rice *ss3a/be2b* mutant, a significantly higher AGPase activity resulting from SSIIIa and SBEIIb deficiency leads to a high concentration of ADP-glucose. GBSSI yields a higher Km for ADP-glucose than other soluble SS isozymes. Coupled with the fact that the GBSSI amounts in *ss3a/sbe2b* and *sbe2b* are significantly higher than those in wild type varieties; these results strongly explain why amylose synthesis is remarkably enriched in the endosperm of *sbe2b* and *ss3a*/*sbe2b* compared with wild-type varieties (Nishi et al., 2001; Asai et al., 2014). In addition, amylopectin synthesis stops at later starch development stage when dehydration begins in *sbe2b*-related mutants, emphasizing the AC in *sbe2b* and *ss3a*/*sbe2b* (Asai et al., 2014).

For other researchers, the third factor is the main one responsible for amylose increase in rice. The *waxy ae* double mutant, which eliminates SBEIIb in an amylose-free background, still contains amylose, and AC increase extent is almost similar to that in its counterpart in *ae* single mutant (Nishi et al., 2001). Thus, amylose increase in the double mutant is not due to the true amylose increase but to modified amylopectin. The maximum wavelength of the absorbance of the starch-iodine complex (λmax) of starch from *waxy ae* is 32 nm higher than that from the *waxy* mutant. Although ELC is not observed directly, this result strongly suggests that the AC increase is caused predominantly by the abnormal structure of amylopectin. Except genetic evidence, structural analyses on debranched starch from two *SBEIIb* down-regulated lines, amiRNA and hpRNA demonstrate directly that AC doubling in these two transgenic lines is not due to an increase in the relative proportion of amylose but to the elevated proportion of ELC, which ranges from degree of polymerization (DP) 120–1,000 (Butardo et al., 2011).

For maize, the amylose increase mainly results from ELC. Although starch contains no amylose, similar to the *waxy ae* double mutant of rice, the *waxy ae* mutant *o*f maize displays an apparent AC of 34.5% (Jane et al., 1999). On the other hand, detailed structural analyses have demonstrated that those long chains do not originate from the real amylopectin of high-amylose starches, but from intermediate components (IC), which is a fraction consisting of branched molecules with molecular weights smaller than amylopectin but similar to amylose (Wang et al., 1993; Kasemsuwan et al., 1995) and is capable of escaping 1-butanol precipitation with amylopectin (Jane and Chen, 1992; Li et al., 2008; Peymanpour et al., 2016). When removing amylose from high-amylose starches, a mixture containing both IC and amylopectin is further separated using gel permeation chromatography (GPC) (Li et al., 2008). Peymanpour et al. (2016) divided this mixture according to their distribution of molecular structure into high molecular weight fraction (HMF) and low molecular weight fraction (LMF). HMF is the typical amylopectin, whereas LMF corresponds to the IC fraction, which increases with AC and appears to have substantially more of long chains than HMF. Li et al. (2008) further divided IC fraction according to the blue-values into large-M_w_ IC and low-M_w_ IC. Chain-length distribution results indicate that large-M_w_ IC has a similar branch structure to amylopectin but with smaller molecular-weight. On the other hand, low-M_w_ IC, which is the main source of fluctuating apparent AC in starch, has longer chains compared with amylopectin and large-M_w_ IC (Li et al., 2008).

## Structure changes of amylopectin in high-amylose cereal crops

In those high-amylose lines, reduced or completely lost SBEs actually does not only result in a simple promotion of AC within the granules, but also complicates the starch structure, specifically the amylopectin structure. Amylopectin is mainly composed of short- to mediated-length chains in normal and waxy cultivars. However, starch from SBEs-deficient lines has an abnormal amylopectin structure, which is enriched with long branch-chains but depleted of short ones (Nishi et al., 2001; Yao et al., 2004). For example, in rice *ae* mutants with the *DP* ≤ 17, chains with DP 8–12 are remarkably reduced. On the other hand, the long branch-chains with *DP* > 24, which connect the clusters of amylopectin, are more abundant compared with those in the wild type varieties (Nishi et al., 2001; Asai et al., 2014). Furthermore, through GPC analyses of a series of different AC-contained starches, Lin et al. (2016c) found that the ratio of long amylopectin branch-chains in high-amylose species is positively correlated with the AC.

Effect of different SBEIIb dosages on the amylopectin in rice is widely researched. First, due to the triploid characteristic of endosperm, Nishi et al. (2001) performed reciprocal crosses between null *ae* mutant (*aeaeae*) and wild type (*AeAeAe*) to generate F1 seeds with two different doses of the SBEIIb allele, duplex (*AeAeae*) and simplex (*Aeaeae*), which shows 74 and 26% protein levels relative to the wild type, respectively. This result implies that the protein level of SBEIIb appears to increase almost linearly with the increase in the number of dominant *Ae* alleles; a similar trend is observed in gene dosage effect on SBEIIb activity. The proportion of short branch-chains with *DP* ≤ 17 is 74% in duplex (*AeAeae*), 72% in simplex (*Aeaeae*), and 66% in nulliplex (*aeaeae*), indicating that *DP* ≤ 17 increases with the increasing number of Ae allele; however, the increase is not linear. Second, when SBEIIb gene is introduced into SBEIIb-defective mutant, resulting transgenic rice plants show a wide range of SBEIIb activities (Tanaka et al., 2004). As the SBEIIb activity increases, short branch-chains with *DP* ≤ 13 become more frequent, whereas the number of long branch-chains with *DP* = 15–30 and *DP* ≥ 40 decline. Although structural and physicochemical properties caused by different SBEIIb dosages are not remarkably increased with the increase of Ae allele or SBEIIb expression level, the above results reveal that starch composition and molecular structure are entirely dependent on the level of SBEIIb activity. In addition, *SBEIIb* overexpression results in a severely shrunken phenotype of the kernel, which is caused by the accumulation of excessive branched, water-soluble polysaccharides instead of amylose and amylopectin (Tanaka et al., 2004).

## Starch properties of high-amylose cereal crops

Increased amylose, especially the enriched percentage of amylopectin long branch-chains dramatically affected starch properties including crystalline structure (Cheetham and Tao, 1998; Nishi et al., 2001; Li et al., 2008; Jiang et al., 2010b; Wei et al., 2010b,d; Butardo et al., 2011; Man et al., 2013a; Asai et al., 2014; Cai et al., 2014c; Lin et al., 2016c), thermal properties (Jiang et al., 2010c; Regina et al., 2010; Wei et al., 2011; Man et al., 2014; Pan et al., 2017), and hydrolysis properties (Jiang et al., 2010d; Qin et al., 2011; Man et al., 2012a,b, 2013b,c; Cai J. et al., 2014; Huang et al., 2015; Lin et al., 2016a). These properties determine the quality of starch and the application of high-amylose crops.

## Crystalline structure of high-amylose starch

Starch is stored as granules with alternating semi-crystalline and amorphous growth rings. The semicrystalline ring consists of the lamellar structure of alternating crystalline and amorphous regions. Amylopectin branch-chains form double helices and are laterally packed to form crystalline regions (Blazek and Gilbert, 2011). Starch crystallinity has types A and B according to X-ray diffraction pattern. A-type crystallinity is formed by amylopectin with short branch-chains and closed branching points, whereas B-type crystallinity is formed by amylopectin with long branch-chains and distant branching points. Usually, waxy and normal cereal crops contain A-type starch (A-type crystallinity), tuberous crops consist of B-type starch (B-type crystallinity), and some legumes and rhizomes possess C-type starch, which is a mixture of both A- and B-type crystallinities (Cheetham and Tao, 1998; Blazek and Gilbert, 2011).

For high-amylose crops with SBEs inactivation, their amylopectin branch-chain length increases and branching degree decreases with increasing AC, leading to the change of starch crystallinity (Table 1). Most high-amylose crop starches display B-type crystallinity (Nishi et al., 2001; Li et al., 2008; Jiang et al., 2010b; Butardo et al., 2011; Asai et al., 2014; Lin et al., 2016c), which is easily understood through the change in amylopectin structure. However, some high-amylose crops have C-type starch (Cheetham and Tao, 1998; Wei et al., 2010a,d; Butardo et al., 2011; Cai et al., 2014c; Huang et al., 2015; Lin et al., 2016c). Cheetham and Tao (1998) proposed that starch changes from A-type to B-type via C-type as AC increases in maize. The transition occurs at approximately 40% AC, a value capable of maintaining C-type starch. Starch with AC lower than 40% has A-type crystallinity, whereas AC higher than 40% has B-type crystallinity. Cai et al. (2014c) thought that high-amylose maize starch with AC 35.6% and apparent AC 56% contain differently sized granules and large and small granules exhibit A- and B-type crystallinities, respectively. However, how and where these different-sized starch granules are synthesized in maize endosperm remains unclear. High-amylose rice TRS with 60% AC is composed of polygonal, aggregate, elongated, and hollow granules distributed in specific endosperm regions (Wei et al., 2010c; Cai et al., 2014b). Polygonal and elongated granule has A- and B-type crystallinity, respectively. However, the aggregate granule contains both A- and B-type crystallinities within the same granule (Wei et al., 2010a,b; Man et al., 2014). TRS is a good material to investigate the synthesis of different crystallinities in the future.

## Thermal properties of high-amylose starch

For the applications of starch in food industry, heating starch in water is necessary. During heating, starch granules absorb water and swell, crystalline structure is destroyed, and birefringence is lost. This process is defined as starch gelatinization and can be detected by hot-stage microscopy and differential scanning calorimetry (Cai et al., 2014a). AC and amylopectin structure in starch granule significantly affect starch gelatinization (Kaur et al., 2007; Wei et al., 2011; Qin et al., 2012; Cai et al., 2014d). Amylose can maintain the integrity of swollen granule and restrain its swelling (Tester and Morrison, 1992). Granule swelling is positively correlated to amylopectin short branch-chains, whereas negatively to amylopectin long branch-chains (Salman et al., 2009; Lin et al., 2016a). In addition, lipid content in starch granule is positively correlated with AC, and lipid-complexed amylose chains also restrict granular swelling (Tester and Morrison, 1992). Therefore, high-amylose starches have lower granule swelling.

For gelatinization temperature, normal starches usually show a clear gelatinization transition peak due to the dissociation of short-chain double-helical crystallites of amylopectin molecules (Jiang et al., 2010c). However, high-amylose starches yield two thermal transition peaks that correspond to the melting of amylopectin double-helical crystallite and amylose-lipid complex (Jiang et al., 2010c; Qin et al., 2012; Man et al., 2014). In high-amylose starch with relatively low AC increase, the amylopectin has longer branch-chains and its double-helical crystallite requires a higher gelatinization temperature than normal starch does; on the other hand, high AC causes the simultaneous formation of an amylose-lipid complex resulting in the second thermal transition peak appearance (Jiang et al., 2010c; Qin et al., 2012; Man et al., 2014). As AC continually increases, the molecular structure of amylopectin is severely disordered; thus, intensity of the first peak gradually becomes weak, whereas the second peak becomes strong (Regina et al., 2010; Man et al., 2014). When AC becomes the dominant component in the starch granules, the first peak disappears and only the second one is preserved (Jiang et al., 2010c; Regina et al., 2010; Man et al., 2014).

## Hydrolysis of high-amylose starch

Starch hydrolysis by amylase is involved in many biological and industrial processes (Tawil et al., 2011). Usually, amylase hydrolyzes starch in two ways as follows: Amyloglucosidase erodes the outer surface of the granule (exocorrosion), and α-amylase creates channels leading to the granule center and consequently leads to its breakdown from the inside out (endocorrosion) (Li et al., 2004). The susceptibility of starch to amylase is influenced by many factors, such as granule morphology, size, integrity and porosity, AC, and crystalline structure (Blazek and Gilbert, 2010). Generally, large starch granule with low relative surface area is slowly hydrolyzed compared with a small granule (Kim et al., 2008). Surface pores and internal channels of starch granule can increase enzyme diffusion into the interior of granule (Naguleswaran et al., 2011, 2012). B-type starch has higher resistance to amylase than A-type starch; amylose inhibits starch hydrolysis (Jiang et al., 2010d; Man et al., 2013b,c; Lin et al., 2016a,b). Proportion of the elongated and aggregate starch granules in high-amylose maize and rice increases with the increase in AC. Elongated and aggregate granules, consisting of two or many fused sub-granules, have larger size than normal starch, show smooth surface without porosity and internal channel, and exhibit high amylose in the outer layer of the granule (Jiang et al., 2010a,d; Wei et al., 2010c; Cai et al., 2014d). Therefore, B-type crystallinity, large size, and smooth surface increase the resistance of high-amylose starches to amylase hydrolysis, and the outer region of the granule is highly resistant to hydrolysis (Jiang et al., 2010d; Man et al., 2013b,c).

## Heterogeneity of starch granules in high-amylose cereal crops

Recently, a highly diverse population of differently stained and different morphological starch granules are detected in the endosperm of high-amylose cereal crop (Jiang et al., 2010a; Wei et al., 2010c; Butardo et al., 2011; Wellner et al., 2011; Carciofi et al., 2012; Liu et al., 2013; Cai et al., 2014d; Man et al., 2014). More interestingly, different morphological granules have an obviously regional distribution in mature kernels (Wellner et al., 2011; Liu et al., 2013; Cai et al., 2014b).

## Occurrence of differently stained starch granules

Isolated starch granules from high-amylose maize and rice grains show different colors under normal and polarized light when being stained with iodine (Cai et al., 2014b,d). More interesting, in the kernel of maize *ae* mutants, a narrow band of sub-aleurone is occupied by blue-staining granules, whereas the central endosperm region is dominated by pink-staining granules. Between the differently stained regions, a transition region with a range of biphasic granules containing blue- and pink-stained colors simultaneously are detected (Liu et al., 2013). This observation indicates that the three differently stained starch granules exhibit a spatial distribution and that the heterogeneity of starch granules increases slightly from the inner to outer layer. Raman microscopy reveals that the pink regions contain starch molecules with reduced levels of branching compared with those blue-stained regions, which are ordered (crystalline) but not radially oriented (Wellner et al., 2011). Thus far, the physicochemical and functional properties of the three differently stained granules have yet to be extensively investigated.

## Occurrence of different morphological starch granules

Recently, several reports state that high-amylose crops have different morphological starch granule distinguished from the normal ones. Normal maize contains angular or spherical granules, whereas in the single mutant *ae*, a small percentage (~1.7%) of elongated granules is observed. Furthermore, this kind of granules has a positive relationship with AC. The percentage of elongated granules is increased to 32% in GEMS-0067 endosperm with 85% AC (Jiang et al., 2010a). Some aggregate starch granules are also reported in high-amylose maize (Cai et al., 2014d). Wheat and barley grains have large lenticular starch granules and small spherical ones; however, the large granules from their high-amylose counterparts become sickle-shaped (Regina et al., 2006, 2010). In barley amylose-only line, multi-lobed starch granules with elongated, rough, and globular morphology dominates the whole kernel (Carciofi et al., 2012). Rice grain is filled with homogeneous compound granules, which can be easily separated to individual polygonal ones with various angles during starch isolation process. In different source of high-amylose rice, including *ae* and Goami 2 mutants and *SBEIIb*-inhibited lines, polygonal granule is mixed with large voluminous, nonangular rounded bodies (Nishi et al., 2001; Kang et al., 2003; Butardo et al., 2011). In high-amylose rice TRS, mature kernel has four types of different morphological starches including polygonal, aggregate, elongated, and hollow granules (Wei et al., 2010c; Cai et al., 2014b).

Even though different morphological starch granules are popularly detected in high-amylose cereals, their physicochemical properties still remain unknown due to the fact that they are always studied as a mixture with the normal ones. Therefore, all the results from high-amylose starch are averaged values. Actually, not only do starch granules with different morphologies have the different structures, but also they contain different physicochemical properties across identical and different species. For example, in wheat and barley, the large granule has higher AC, gelatinization enthalpy and pasting viscosity, and lower amylopectin short branch-chains and swelling power than its small counterpart, which results in different end uses (Salman et al., 2009; Naguleswaran et al., 2011; Li et al., 2013). Small starches are predominantly applied as fat substitute, paper coating, and as carrier material in cosmetics, whereas large starches are used to manufacture biodegradable plastic film, carbonless copy paper, and brewing beer (Lindeboom et al., 2004). Thus, separation and purification of different morphological starch granules are essential for further applications in food and non-food industries.

## Research on purified heterogeneous starch granules from high-amylose rice TRS

Contrary to the distribution of different morphological starch granules in the grain of *ae* maize mutants (Liu et al., 2013), the heterogeneous starch granules with polygonal, aggregate, elongated, and hollow shapes in high-amylose rice TRS grain have obvious regional distribution from the inner region to the outer region. The polygonal starch is located in the innermost region, the aggregate, and elongated starches exist in the center region, and the hollow starch is distributed in the outmost region (Cai et al., 2014b). To investigate their individual structure and physicochemical properties, the four granule types are separated and purified on the basis of their regionally distributed characteristics (Man et al., 2014). GPC analysis shows that from the polygonal to hollow starch granules, their AC displays a gradually elevated trend, from 35.9 to 75.8%. The four granule types are essentially different in relative crystallinity, short-range ordered structure, relative proportions of single helix, double helix, and amorphous conformation; these differences imply that their structures are completely divergent (Man et al., 2014). The different structures of TRS heterogeneous starch granules yield significantly different XRD patterns, thermal properties, and digestion properties. Polygonal starch exhibits A-type crystallinity, aggregate and elongated starches show C-type crystallinity, whereas hollow starch has no crystallinity (Man et al., 2014). Cai et al. (2014b) found that polygonal and elongated starches have the lowest and highest gelatinization temperatures, respectively, while the gelatinization temperature of hollow starch is undetected due to its extremely weak or no birefringence. The *in vitro* digestion properties of native starches from the four granule types are investigated, and results reveal that polygonal and aggregate starches possess monophasic digestograms, whereas elongated and hollow starches exhibit biphasic digestograms from 0 to 8 h mainly because of their different structural compositions (Huang et al., 2016).

## Conclusions and future perspectives

In maize and rice, mutating or suppressing *SBEIIb* is necessary to obtain high-amylose lines. However, the efficiency for an AC increase in rice is much lower than that in maize. When HAM genes are introduced to maize *ae* mutants and SBEI and SBEIIb are simultaneously inhibited in *indica* rice, AC is further increased and thus reaches 88.2 and 60%, respectively. In wheat and barley, high-amylose lines with AC (>85%) are derived by simultaneously suppressing *SBEIIb* and *SBEIIa*. The enhanced amylose synthesis and the suppressed amylopectin synthesis and the ELC from amylopectin/IC are responsible for AC increase in high-amylose species. Along with the AC increase, fewer longer branch-chains are detected in amylopectin. The significantly altered structure of high-amylose starches causes a series of other important changes, such as crystallinity, thermal properties, and hydrolysis properties.

Different morphological starches and differently stained granules are detected in high-amylose rice and maize. They are also regularly distributed in the grains from the inner to outer regions and occupy different molecular structures. Four heterogeneous granules in high-amylose rice TRS possess different physicochemical and digestion properties, which can be used for further applications in food and non-food industries. Future biochemical experiments should be performed to elucidate how heterogeneous starch granules are formed in one kernel. The different morphological starches are also detected in other high-amylose cereal crops, but whether these heterogeneous starches exhibit regional distribution in kernels and have different physicochemical properties are unknown.

Studies have also yet to identify the causes of regional distribution of heterogeneous starches in kernels. Grain filling originally begins in the core and then spreads to the outer region during endosperm development in rice and maize. Combined with regionally distributed phenotypes in high-amylose grains, various regulated mechanisms may be observed in different regions of grains. For example, white-core mutants, such as *flo4, flo5*, and *rsr1* (Kang et al., 2005; Ryoo et al., 2007; Fu and Xue, 2010), and aberrant-periphery mutants, including *flo7* (Zhang et al., 2016), play essential roles in the early stage and late stages of endosperm development in rice, respectively. However, we have yet to reveal how starch synthesis-related enzymes are regulated in grain during endosperm development from the inner parts to the outer parts.

## Author contributions

All authors listed, have made substantial, direct and intellectual contribution to the work, and approve it for publication.

### Conflict of interest statement

The authors declare that the research was conducted in the absence of any commercial or financial relationships that could be construed as a potential conflict of interest.
